# Depression predicts future emergency hospital admissions in primary care patients with chronic physical illness

**DOI:** 10.1016/j.jpsychores.2014.10.002

**Published:** 2016-03

**Authors:** Elspeth A. Guthrie, Chris Dickens, Amy Blakemore, Jennifer Watson, Carolyn Chew-Graham, Karina Lovell, Cara Afzal, Navneet Kapur, Barbara Tomenson

**Affiliations:** a Manchester Mental Health and Social Care Trust, Rawnsley Building, Manchester Royal Infirmary, Manchester, UK; b Institute of Health Service Research, University of Exeter Medical School, Exeter, UK; c Research Institute, Primary Care and Health Sciences, Keele University, Keele, Staffordshire, UK; d School of Nursing, Midwifery and Social Work, The University of Manchester, Room 6.322a, Jean McFarlane Building, University Place, Oxford Road, Manchester, UK; e Centre for Suicide Prevention, University Place, The University of Manchester, Oxford Road, Manchester, UK; f Biostatistics Unit, Institute of Population Health, The University of Manchester, Jean McFarlane Building, Oxford Road, Manchester, UK; g Peninsula Collaboration for Leadership in Health Research and Care (PenCLAHRC), University of Exeter, Veysey Building, Room 007, Salmon Pool Lane, Exeter, UK; h National Institute for Health Research School for Primary Care Research, Centre for Primary Care, Institute of Population Health, Manchester Academic Health Science Centre, University of Manchester, Manchester, UK

**Keywords:** Depression, Chronic physical illness, Chronic physical illness, Urgent care, Hospital admission, Primary care

## Abstract

**Objective:**

More than 15 million people currently suffer from a chronic physical illness in England. The objective of this study was to determine whether depression is independently associated with prospective emergency hospital admission in patients with chronic physical illness.

**Method:**

1860 primary care patients in socially deprived areas of Manchester with at least one of four exemplar chronic physical conditions completed a questionnaire about physical and mental health, including a measure of depression. Emergency hospital admissions were recorded using GP records for the year before and the year following completion of the questionnaire.

**Results:**

The numbers of patients who had at least one emergency admission in the year before and the year after completion of the questionnaire were 221/1411 (15.7%) and 234/1398 (16.7%) respectively. The following factors were independently associated with an increased risk of prospective emergency admission to hospital: having no partner (OR 1.49, 95% CI 1.04 to 2.15); having ischaemic heart disease (OR 1.60, 95% CI 1.04 to 2.46); having a threatening experience (OR 1.16, 95% CI 1.04 to 1.29); depression (OR 1.58, 95% CI 1.04 to 2.40); and emergency hospital admission in the year prior to questionnaire completion (OR 3.41, 95% CI 1.98 to 5.86).

**Conclusion:**

To prevent potentially avoidable emergency hospital admissions, greater efforts should be made to detect and treat co-morbid depression in people with chronic physical illness in primary care, with a particular focus on patients who have no partner, have experienced threatening life events, and have had a recent emergency hospital admission.

## Highlights

• Depression significantly predicts prospective emergency hospital admissions over 12 months.
• Severe depression increases the likelihood of emergency hospital admissions by more than two fold.
• Emergency admission in the previous 12 months increases the risk of admission by 3 and a half times.
• Having no partner, heart disease and threatening experiences also predict emergency admissions.

## Introduction

Healthcare systems in the Western world are struggling to cope with the increasing burden of people with chronic physical health conditions, such as diabetes, arthritis, asthma or cardiovascular disease. More than 15 million people in England (30% of the population) have one or more chronic physical diseases and approximately 70% of the entire healthcare budget in England is spent on chronic physical disease [1]. There is a similar pattern in the United States where over 80% of all healthcare spending was spent on the 50% of the population who have a chronic medical condition [2], [3].

Most people with a chronic physical health problem have more than one chronic disease [1], [4], [5], [6], and multimorbidity, defined as the co-existence of two or more chronic conditions [7], is associated with increasing age and high rates of social deprivation [8]. As life expectancy increases in the West, chronic, multimorbid physical illness will become even more prevalent [1], [9].

Research evidence consistently demonstrates that people with chronic physical illness are two to three times more likely to experience depression than the general population. Depression is common in a range of cardiovascular diseases including heart failure, coronary artery disease, stroke, angina and post myocardial infarction [10], [11]. People living with diabetes are between 1.5 to 3 times more likely to have depression than the general population and the rates of depression [12], [13] may even be higher in people suffering from chronic obstructive pulmonary disease [14].

Comorbid depression in chronic physical disease is linked to a series of poor outcomes [15], including increased morbidity [9], [16], [17], [18], mortality [19], [20], and greater healthcare utilisation [11], [20], [21], [22]. For example in diabetes comorbid depression is associated with poorer glycaemic control, more diabetic complications, increased mortality and increased risk of dementia [17], [23].

An analysis of USA national claims data for more than nine million people showed that patients with chronic physical disease who were also receiving treatment for depression or anxiety had average monthly medical costs that were between 33% and 169% higher over a range of conditions. These costs excluded expenditure on mental health services [24].

As costs rise, the spotlight has focused upon unscheduled care costs in people with chronic physical disease. In some conditions this can amount to up to 50% of the total health care costs [25]. It is possible that some use of unscheduled care is preventable if there was greater understanding of the factors that contributed to its use [26]. In addition to health factors, recent research evidence suggests that depression is associated with increased use of urgent care in people with chronic physical disease.

A recent systematic review of 16 prospective studies of patients with chronic physical illness found that depression increased the risk of using urgent care by approximately 50% [27]. However, the findings were qualified, as only half of the studies were able to control for severity of illness, and very few considered multimorbidity. In addition, most of the studies were hospital based, and focused on different sub-groups of patients (e.g. people admitted to hospital following an exacerbation of COPD). Only two focused on patients in a primary care setting [28], where most people with chronic physical illness are managed, and where any potential preventable intervention is likely to be delivered.

Of the primary care studies, one was based in the USA and followed 367 patients with diabetes over a one year period [28], and the other was based in Germany and followed 256 patients with asthma over a 12 month period. In both studies depression predicted greater attendances at the emergency department in the prospective year, independent of HbA1C in the former study and independent of asthma severity in the second study.

Life stress is increasingly recognised to play important roles in the development and outcomes of chronic illness such as type II diabetes, coronary heart disease and COPD [29]. Stress is well known to lead to depression, and chronic illness may result in greater stress (e.g. financial problems because of not being able to work), setting up a vicious circle. Thus it may be important to try to disentangle the role of life stress from that of depression in any study of psychosocial factors in chronic physical disease.

The aim of this prospective study was to determine whether depression is associated with future emergency admission to hospital in primary care patients with chronic physical disease.

## Method

We conducted a prospective cohort study of adult patients with chronic physical disease in primary care. We focused on four exemplar, common physical conditions that are easily identifiable from patient registers kept by general practices in England under the Quality and Outcomes Framework (QOF) [30]: diabetes, ischaemic heart disease (IHD), chronic obstructive pulmonary disease (COPD) and asthma. It was not feasible to study every chronic physical condition. The four diseases that we chose represent 3 of the top 4 most burdensome non-communicable diseases worldwide [31], have been shown to be among the most common discharge diagnoses from emergency departments [32] and have been associated with emergency hospital admissions [33].

All patients with at least one of the four exemplar conditions were identified from QOF registers in 10 general practices in inner city Manchester, England. A variety of strategies were used to recruit practices including direct invitation and presentations at local meetings. Following this we contacted 31 practices informally by telephone to discuss the study in more detail, and then wrote to 21 practices formally inviting them to participate, of which 10 agreed. No incentives were given to practices to participate in the study, although they were provided with support costs to cover the work that they carried out in relation to the study.

Once a practice had agreed to support the study, lists of potentially eligible patients were checked by practice GPs, who excluded patients receiving palliative care, or who were not thought to have capacity to complete questionnaires. Eligible patients were sent a postal questionnaire between June 2010 and December 2010, with a further reminder questionnaire pack 3 weeks later. We used the following strategies to maximise response [34]: a personalised letter addressed to the individual from their GP; an explanation which focused upon the importance of both physical health and mental health; coloured ink; stamped addressed envelopes; and an explanation that the research was funded by the NHS and conducted by the university as opposed to a commercial organisation. We also offered translation services for participants who wished to complete the questionnaire in a language other than English. Eligible patients who did not wish to participate were asked to return their questionnaires blank in the stamped addressed envelope that we provided. At baseline blank questionnaires were returned to the general practice and at follow-up they were returned to the research team. Participants were asked to give permission for review of their medical records for one year prior to the date of completion of the questionnaire and for the year of the prospective study period.

### Sample size

This was based on the percentages of patients who had used urgent care being 15% in the group without a risk factor compared with 30% in the group with that risk factor (odds ratio = 2.43). The study would have 90% power to detect a difference at the 5% level with sample sizes of 400 with and 100 without the risk factor. The study aimed to receive completed questionnaires from at least 500 patients for each of the four chronic illnesses.

## The postal questionnaire data

### General data

Age, gender, ethnicity, marital status, current work status, education level, other physical illness (arthritis/joint problems, high blood pressure, stomach/bowel problems and cancer), and distance from home to the nearest emergency department (calculated using each respondent's postal code) are included.

### Depressive symptoms

The *Hospital Anxiety and Depression Scale* (HADS) was used [35]. In this study, we focused on the depression subscale which has 7 items with a maximum score of 21. This measure was originally developed for use with patients with physical illness and does not include somatic symptoms of depression. Such symptoms are common in physical disease states, so questionnaires to measure depression that include such items may overestimate depression in populations with physical disease [36]. We used a cut off of 8 to identify patients with depressive symptoms but we also divided the scale scores into quintiles to examine the individual effects of different severities of depression [20]. The highest quintile was a score of 11 or more, which has been used as an indicator of a probable depressive disorder in people with physical disease [37].

### Recent stress

The *List of* *Threatening Experiences* (LTE-Q) measures the experience of 12 threatening personal situations or events in the last 6 months [38]. The total score of positive responses represents recent exposure to threatening experiences. We excluded from the total the item “serious illness or injury to subject”, as it may have related to the patient's chronic physical illness.

## GP record data

### Severity of chronic physical disease

The severity of each of the four chronic physical diseases was collected from respondent's medical records and each condition was then independently rated as being mild, moderate, severe or very severe. Following extensive discussion with the study team, the following were determined as the best way of categorising severity, based upon the likely information available in the GP records.

COPD severity was classified using the FEV1 (forced expiratory volume in 1 s) percentage predicted values for the patient age, height and sex as recommended by the Global Initiative for Lung Disease [39]. Asthma severity was classified according to the intensity of the treatment that was required to achieve good asthma control as recommended by the Global Strategy for Asthma Management and Prevention [40]. CHD severity was classified using the New York Heart Association classification (1994) [41] which categorises patients based on how much they are limited during physical activity; the limitations/symptoms are in regard to normal breathing and varying degrees in shortness of breath and or angina pain. The most severe score requires patients to have symptoms whilst at rest.

Diabetic severity was classified according to a proxy measure, glycosylated haemoglobin levels (HbA1c), with 0–6.4% = mild, 6.5%–7.4% = moderate, 7.5%–8.4% = severe and 8.5% or more = very severe. HbA1c scores are associated with more severe disease and complications [42], and this data is more reliably recorded in the GP records than that for diabetic complications. If patients had more than one condition, the maximum severity and total severity scores using 0 = none, 1 = mild, 2 = moderate, 3 = severe, and 4 = very severe were also derived for each patient. Thus the total severity score had a maximum possible range from 0 to 16.

## Emergency hospital admissions

The GP notes were also used to identify the number of emergency in-patient hospital admissions for the year before the date of completion of the questionnaire and the year following, for respondents who gave consent for their medical notes to be checked.

The study received ethical approval from the Northwest 8 Research Ethics Committee — GM East Reference: 09/H1013/80. All participants provided written informed consent. We followed the STROBE guidelines for reporting observational studies [43].

## Statistical analysis

For all demographic variables, questionnaire scores, and hospital admission data we present numbers and percentages for categorical variables, and mean scores with standard deviation for continuous variables. Respondents who had an emergency admission in the follow-up year were compared with those who did not using t-tests for scores and either chi-squared test or Fisher's exact test for categorical variables, as appropriate.

Logistic regression was used to assess the relationship between baseline variables and emergency admission to hospital in the prospective year. All baseline variables were entered, with depression entered using a cut-off of 8 to define caseness. Two separate analyses are presented: model 1 does not include data on emergency admissions in the preceding year, and model 2 does include this. We expected previous admission to be a strong predictor of future admission and therefore the addition of previous admission as an independent variable in model 2 was to determine whether other significant risk factors remained unchanged after previous admissions were accounted for. Odds ratios and 95% confidence intervals are presented for all baseline variables found to be significantly associated with emergency admission in the prospective year. Variations on the logistic regression model were also carried out, replacing the 4 individual chronic diseases by the number out of 4.

HADS depression scores were then split into 5 quintile groups of approximately equal sample size, as follows: 0–1, 2–4, 5–7, 8–10, and 11 or more. Logistic regression was used to assess the association between the five depression quintile groups and emergency hospital admission during the prospective study period. Unadjusted odds ratios are presented for each quintile group with the lowest group as reference group. The analysis was then repeated using logistic regression to adjust for all relevant covariates, identified from the initial analyses described in the previous paragraph. These included age, sex, marital status (single, widowed, separated or divorced versus married or cohabiting), poor education, not working due to ill health, presence of each of 4 QOF conditions, presence of cancer, stomach problems, high blood pressure, arthritis, number of threatening experiences, distance from the nearest hospital, the maximum severity of the QOF diagnoses, and urgent hospital admission during the baseline period.

Since the data collected from the various sources may be biased by non-completion of questionnaires, and/or non-availability of medical notes, we have used inverse probability sampling weights using all the data which was available to us to adjust for this potential bias in all the multivariate analyses. For questionnaire data, these were calculated using the reciprocal of the probability of completion, based on age group, gender and GP practice (1860 returned out of 6692 eligible). Additional inverse probability sampling weights were calculated for lack of permission to review, and unavailability of, GP notes data, and the product of the two weights was used for all analyses on data extracted from GP notes. Stata imputation was used to impute values for missing data on the independent variables. Multicollinearity was not a problem as the largest Variance Inflation Factor (VIF) was 1.9. Analyses were carried out using IBM SPSS version 20 and Stata version 12.1 (StataCorp LP, Texas, USA).

## Results

**Fig. 1:**
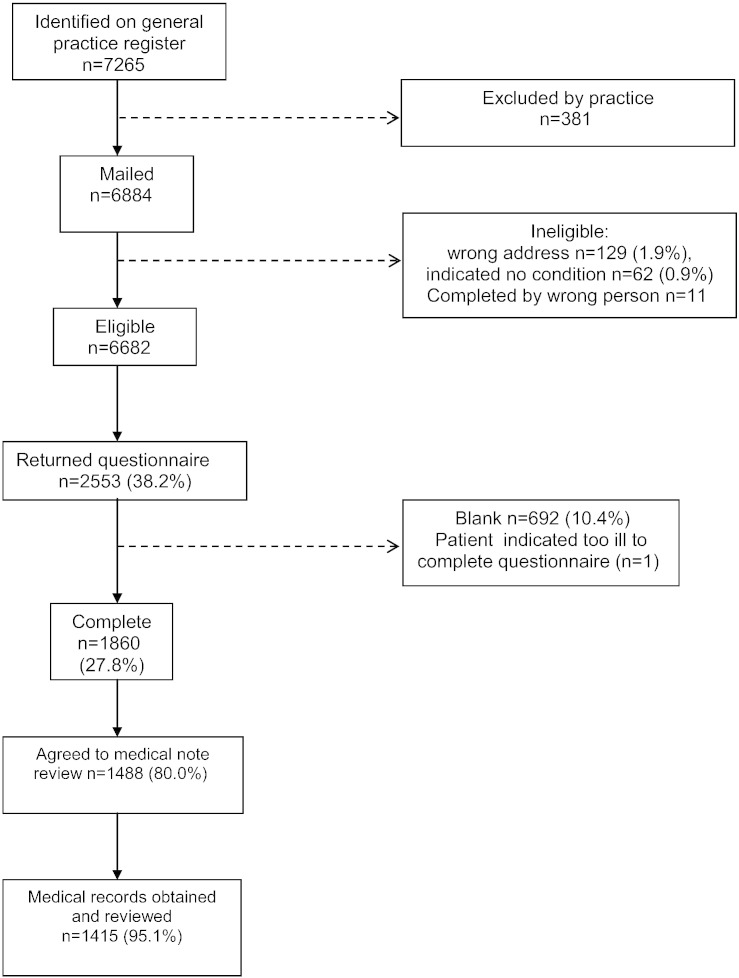
Flow of study participants.

The flow of participants to the study is shown in Fig. 1. We invited 31 general practices to take part in the study, and 10 practices accepted (32.3%). Based on the 2010 condition prevalence rates for CHD, COPD, diabetes and asthma by practice there were no significant differences between those who took part and those that did not. Questionnaires were sent to 6682 participants (Fig. 1) with 2553 responding (38.2%). Of those returned, there were 1860 usable questionnaires (27.8%). 1488 patients (80%) provided consent for their medical records to be reviewed, and these were retrieved and examined for 1411 patients for the year before and 1398 patients for the prospective year.^1^ Patients whose notes were examined were significantly more likely to be male (*P* = .026) and to have reached a moderate educational standard (i.e. some ‘O’ levels, GCSEs or higher) (*P* = .002) than patients whose notes were not examined. Severity was available for 295 out of 465 (63.4%) CHD, 469 out of 523 (89.7%) asthma, 417 out of 465 (89.7%) diabetes and 228 out of 355 (64.2%) COPD patients.

More women responded than men (28.6% versus 25.6%, *P* = .007), and more older patients responded than younger patients (response rate: 36.0% for patients aged 70 to 79, decreasing to 9.9% for patients aged 18 to 29, but 30.1% for patients aged 80 or more, *P* < .001). Response rates ranged from 16.7% to 35.2% at the 10 different practices (*P* < .001). There were no significant differences on any variables between the patients who returned the questionnaire without prompting and those who returned it after receiving a reminder (467 (25.1%)). Eight out of the ten practices were in the top 10% of most deprived areas in England according to the Index of Multiple Deprivation (IMD), with 5 in the top 5% and 2 in the top 1% [44]. A higher IMD score indicates greater deprivation across 7 dimensions including: employment, health and disability, education, crime, housing, services and living environment. The 10 practices that took part in the study had significantly higher IMD scores (n = 10, median = 56.23) than those practices that did not take part in the study (n = 21, median = 42.20, U = 57.00, Z = − 2.02, *P* < .04).

We achieved our pre-determined sample size (n = 500) for all of the 4 chronic diseases except for COPD. Out of the 1860 patients who returned questionnaires the QOF diagnoses from the GP databases were as follows: 590 had IHD, 708 had asthma, 617 had diabetes and 449 had COPD. There were 963 females in the cohort (51.8%), the mean age was 62.3 years (SD = 15.4) and 81.6% identified themselves as white British.

In addition to the 4 exemplar chronic diseases, patients self-reported a wide range of other co-morbid medical conditions including arthritis (43.3%, n = 805), hypertension (38.5%, n = 717), stomach/bowel problems (15.4%, n = 287) and cancer (4.4%, n = 81). There was considerable multimorbidity with over 20% of patients having at least 2 of the 4 exemplar diagnoses, and 65.3% (n = 1214) having at least one of the exemplar diagnoses plus another self-reported LTC.

A total of 1818 respondents completed the HADS, of whom 39.6% scored 8 or more (95% CI 37.4% to 41.9%) and 20.7% scored 11 or more (95% CI 18.8% to 22.5%) for depression. The range of scores was 0 to 21 (mean = 6.5, standard deviation = 4.7).

### Emergency hospital admissions during study baseline and prospective periods

Of the baseline cohort of 1411 patients, whose GP notes were reviewed, 221 (15.7%) had an emergency admission in the year prior to completing the questionnaire. During the prospective follow-up period of 12 months, of 1398 GP records, 234 (16.7%) patients had at least one emergency admission to hospital.

Univariate analyses showed that having an emergency admission in the year prior to completion of the questionnaire was associated with: older age (*P* < 0.001); being widowed, separated or divorced (*P* = 0.005); low level education (*P* < 0.001); HADS score of 8 or more (*P* = 0.001); COPD (*P* = 0.018); CHD (*P* = 0.001); self-reported cancer (*P* = 0.021); stomach or bowel problems (*P* = 0.016); arthritis and/or joint problems (*P* = 0.022); and living closer to an emergency department (*P* < 0.001). Patients with diabetes (*P* = 0.024) or asthma (*P* = 0.048) were significantly less likely to have had an emergency admission than the rest of the patients in this study.

Having an emergency hospital admission in the follow-up year was significantly associated with older age; being widowed, separated or divorced; low level education; HADS depression score of 8 or more; COPD or CHD, but not asthma; self-reported arthritis and/or joint problems; more severe physical illness; experiencing a threatening life event; and an emergency admission to hospital in the previous year (Table 1). Although the percentage of patients with an emergency admission at the 10 practices in the follow-up year ranged from 3% to 27%, this was not significant (X^2^ = 15.2, df = 9, *P* = 0.085).

In logistic regression the baseline variables which were significantly independently associated with prospective emergency hospital admissions were: having no partner, IHD, reporting a threatening life experience, an emergency admission to hospital in the previous year and a HADS depression score of 8 or more (Table 2, model 2). The remaining results were very similar even when emergency admission to hospital in the previous year was not included (model 1, Table 2).

### Sensitivity analyses

When the logistic regression analyses were repeated using the total number of the 4 exemplar chronic diseases instead of the 4 individual conditions, the same risk factors were identified with very similar results as in Table 2, and the number of chronic diseases was significant (OR = 1.38, 95% CI 1.02 to 1.88, *P* = 0.040). The odds ratios (and 95% confidence intervals) for the other risk factors were 1.49 (1.04 to 2.13) for lack of partner, 1.16 (1.04 to 1.29) for number of threatening experiences, 3.38 (1.99 to 5.74) for having had an emergency admission in the previous year and 1.69 (1.14 to 2.49) for HADS depression score of 8 or more.

Using the 5 significant independent variables from Table 2, a risk score was calculated for each patient, which ranged from 0 to 5. Fifteen out of 157 patients with none of the risk factors (9.6%) had an emergency admission in the prospective year, compared with 8.0% for any 1, 17.9% for any 2, 24.7% for any 3, 28.6% for any 4 and 30.8% for all 5 risks.

Having had a prior emergency admission to hospital was associated with the greatest risk of future admission to hospital (positive predictive value = 32.4%, sensitivity = 30.3%, specificity = 87.2%). The PPV means that 32.4% of the patients who had an emergency admission in the previous year had another in the prospective year. Out of 219 patients who had an emergency admission in the previous year, 71 (32.4%) had one in the follow-up year, compared with only 13.9% (163 out of 1175) patients who did not have an emergency admission in the previous year. However, for patients who had not had an emergency admission in the previous year, the other 4 factors combined had a positive predictive value of 25.9%, sensitivity = 9.2%, and specificity = 95.8%.

### Depressive symptoms

To determine whether a HADS cut-off of 8 or higher was appropriate we divided the HADS depression scores into quintiles [20]. Worsening depression scores were associated with an increased risk of an emergency hospital admission, with a score of 11 or more on the HADS depression scale more than doubling the risk of requiring an emergency hospital admission in the prospective year compared with, after adjusting for all relevant covariates (Table 3).

## Discussion

This is a large longitudinal prospective cohort study of patients with chronic physical illness in primary care in the UK, which has examined the relationship between depression and risk of future emergency admission to hospital for a physical illness. We found that baseline depression was a significant predictor of prospective emergency hospital admissions over a 12 month period, after controlling for potential confounders including demographics, physical disease multi-morbidity, severity of illness, and previous hospital emergency admissions. The highest severity of depression increased the likelihood of emergency hospital admissions by more than two fold.

Unsurprisingly, a history of emergency hospital admission in the year prior to completion of the questionnaire was the strongest predictor of emergency hospital admission in the prospective study period, increasing the risk by three and a half times. Other factors that were independently associated with an increased risk of emergency hospital admission were: having no partner, having ischaemic heart disease and experiencing an increasing number of threatening life events. Although the contribution of threatening life events appeared small from the regression model, the risk presented is for each additional item on the scale, so an individual who had experienced several threatening life events, would have an increasing incremental risk of hospital admission.

Patients who had all 5 factors had a 43% probability of having an emergency hospital admission during the following year; a nearly 1 in 2 chance. Even in patients who have not had an emergency admission, the other 4 factors in combination had a 1 in 4 chance of prospectively identifying patients who would go on to have an emergency admission in the follow-up year. This was in marked contrast to the patients at baseline who had none of the five risk factors, in whom there was less than a 1 in 10 chance of an emergency hospital admission in the follow-up year.

The severity of physical illness and severity of multimorbidity were both significantly associated with an increased risk of prospective emergency hospital admission in the univariate analyses, as would be expected. However, the effects of these factors were less evident in the final regression model. This may be because all the patients in the study had at least one chronic condition, and we had to enter the diagnoses separately and then in a combined format. However, the variable ‘number of diagnoses’ only just failed to reach significance for the final model, and it is well established that depression and severity of disease are closely related to each other, as is multimorbidity [9]. Whilst there are undoubtedly interactions between all three, the results of this study suggest that depression has a powerful effect on urgent hospital admission, which is independent of the other two. Ischaemic heart disease was also an independent predictor and this suggests that certain chronic diseases carry greater risks of using urgent care than some others.

Much effort has been devoted in the UK to find algorithms that can identify patients at risk of hospital admission, and such models have tended to focus upon those patients deemed to be at the highest risk [45]. There are two major problems however, with this approach. First, those deemed to be at the highest risk account for only a small proportion of overall admissions [46] and second, the likelihood of readmission to hospital in such patients falls with each subsequent year [47].

In this study, we have adopted a hybrid approach to this problem. Instead of trying to identify risk factors for all patients, we have focused upon a sub-group with known physical health problems and who are already under review by GPs, because of their chronic physical illness. We have broadened the baseline parameters to include psychosocial variables, which are almost exclusively missing from most other risk algorithms.

Strengths of this study include the following: a large cohort of primary care patients; recruitment target achieved in 3 out of the 4 exemplar conditions; independent measurement of healthcare which employed scrutiny of primary care records; use of a standardized instrument to measure depressive symptoms; and the independent assessment of severity of illness using primary care records. A further strength was the inclusion of patients with several physical disease conditions as opposed to focusing upon a single disease, with most patients in the baseline cohort having multi-morbidity. The follow-up data of the patients who were entered into the prospective study was good with nearly 80% giving consent for their medical records to be checked.

The response rate, however, to the postal questionnaire was low, but of a similar magnitude to other recent large scale general practice studies utilising self-report measures in the UK: the GP Patient Survey [48] and a recent large primary care cohort study from Oxford which targeted patients with chronic physical disease [49]. The study was also carried out in an area of high deprivation where response rates to postal questionnaires have been falling over the last 15 years, dropping by 20% from 1993 to 2004 [50].

Low participation rates are not necessarily associated with bias [51]. In fact, large variations in participation rates have at most been weakly associated with bias [52]. It is however important to consider the extent to which non-participation in the present study may have biased its outcome.

Participants were more likely to be female and older in age than non-participants. The highest participation rates were for adults between 70 and 79 years of age, and the lowest were for young adults. Therefore our study sample is more representative of older adults.

We were unable to access any other personal information regarding the non-participants in our study, but people with a white British background (82%) were over-represented in our sample when compared with the general population figure of 67% for our 10 general practices [53]. Our study may, therefore, under-represent people from ethnic minority backgrounds with chronic physical disease.

All of the general practices invited to take part in this study were from inner city Manchester, England but those practices that took part had significantly higher levels of social deprivation than those practices that did not. A large retrospective cohort study conducted in Scotland, UK found that when physical and mental health conditions were controlled for the patients who experienced the most social deprivation, they were more likely to have an unplanned hospital admission [54]. This may limit the generalisability of our findings to areas of high social deprivation.

We were unable to control for the reason or cause of hospital admissions in our analyses, as the exact cause of admission is not always clear from the hospital correspondence. However, this is an issue to consider for future research in this area, provided reliable data can be obtained.

A crucial question is whether people with depressive symptoms were over- or under-represented in our study sample. The prevalence rates of depression in the present study are consistent with previous studies which have shown diagnostic rates of depression of approximately 25% in patients with chronic physical disease [55] which are broadly in line with the HADS cut-off of 11 or more, and sub-threshold depression rates of approximately 40–50% [21], which are broadly in line with the HADS cut-off of 8 or more. So it seems unlikely that people with chronic physical illness and depression were neither over- nor underrepresented in the study sample.

We attempted to adjust for differences in age and gender between responders and non-responders using inverse probability sampling weights based on age, gender and practice, which were the only variables available to us for the non-responders. We also compared the responses of patients who returned and completed the questionnaire spontaneously, with those who returned the questionnaire, after a reminder. The latter group could be argued to be more representative of non-responders, as they would not have responded without prompting. There was no difference between these two groups on any of the variables.

Our results cannot be generalized to all populations of patients with chronic physical disease, but they suggest that in a substantial proportion, depression is an important determinant of emergency hospital admission. Our findings also require replication in a separate cohort or equivalent data set. To our knowledge, this is the only UK primary care study which has investigated the relationship between depression and use of urgent care in chronic physical disease.

The proactive management of people with chronic physical disease is a key priority for the NHS in England, and general practice is seen as having a central role in delivering more integrated and personalised care. Not all the variables that we measured are easily available via routine screening, and there is still no clear benefit, even regarding routine screening for depression in primary care [56].

However, we are not aware that the value of screening for key psychosocial variables in certain high risk groups has been fully explored. The qualitative work that we have carried out suggests that depressed patients with chronic physical disease may not themselves recognise that they are depressed and therefore may not ask for help for their mood [57]. Our findings suggest that further evaluation is required regarding the potential benefits of case-finding for depression in chronic physical disease, provided depression can be modified and treated. Without an effective intervention to offer to patients, case-finding will not improve outcome [58], [59].

Current service models are often orientated around single diseases and fail to provide co-ordinated care to the large and growing number of people with combined mental and physical health problems. There is mounting evidence that certain models such as collaborative care may be of benefit [60], [61], but there are challenges to how such models are effectively disseminated and integrated into different healthcare systems [4].

The results of this study suggest that there should be a greater focus on the psychosocial aspects of chronic physical illness, both in terms of identification and treatment, as this has significant implications for patients' use of urgent care.

## Author contributions

Guthrie: planned and designed the study, supervised the research, wrote the first draft of the paper for submission, co-ordinated responses from other authors and produced each iteration of the paper, including the final draft.

Dickens: contributed to the planning and design of the research, was involved in discussing the analysis and was involved and participated in all iterations of the paper.

Blakemore: sent out the postal questionnaires, liaised with the GP practices, helped with data collection from GP records, worked out the severity ratings, contributed to the paper, and approved the final version of the paper.

Watson: sent out the postal questionnaires, liaised with GP practices, co-ordinated the collection of data from the GP notes, worked on the severity ratings, contributed to the paper and approved the final draft.

Chew-Graham: contributed to the planning and design of the study, helped recruit GP practices, contributed to the first draft of the paper and all iterations of the paper and approved the final draft.

Lovell: contributed to the planning and design of the study, contributed to the first draft of the paper and all iterations of the paper and approved the final draft.

Afzal: co-ordinated the sending out of postal questionnaires, liaised with GP practices, contributed to the paper and various iterations and approved the final draft.

Kapur: contributed to study design, contributed to the paper and approved the final draft.

Tomenson: assisted in the design of the study, conducted the statistical analysis, assisted in the drafting of the paper, contributed to all of the iterations, and approved the final version of the paper

## Figures and Tables

**Table 1:** Characteristics of the study participants: those who had an emergency admission to hospital in the prospective year versus those who did not

Demographic variables	Had an emergency admission (n = 234)		Did not have an emergency admission (n = 1164)		Comparison^a^	
N	%	N	%	X^2^	*P*
Female	109	46.6	595	51.1		0.22
Marital status						
Single	37	16.0	219	19.2	12.9	0.002
Married or cohabiting	95	41.1	573	50.1		
Widowed, separated or divorced	99	42.9	351	30.7		
Poor education^b^	150	64.1	657	56.4		0.035
Not working due to ill health	40	17.1	162	13.9		0.22
HADS depression score of 8 or more^c^	113	48.9	415	36.4		< 0.001
HADS depression score of 11 or more^c^	66	28.6	211	18.5		0.001
Medical conditions						
QOF diabetes	86	36.8	375	32.2		0.20
QOF COPD	77	32.9	274	23.5		0.004
QOF asthma	62	26.5	454	39.0		< 0.001
QOF CHD	102	43.6	356	30.6		< 0.001
Self-reported cancer	15	6.4	49	4.2		0.17
Self-reported stomach/bowel problems	47	20.1	177	15.2		0.078
Self-reported high blood pressure	103	44.0	444	38.1		0.11
Self-reported arthritis/joint problems	129	55.1	473	40.6		< 0.001
Severity^d^						
Mild	49	25.9	238	25.2		
Moderate	68	36.0	449	47.6	12.9	0.005
Severe	45	23.8	180	19.1		
Very severe	27	14.3	77	8.2		
Threatening experiences (out of 11)						
None	92	39.3	534	45.9	6.5	0.039
One	54	23.1	289	24.8		
2 or more	88	37.6	341	29.3		
Had an emergency admission in the previous year	71	30.3	148	12.8		< 0.001

Continuous variables	Mean	SD	Mean	SD	t^e^	*P*

Age in years	65.8	14.0	61.4	15.3	4.1	< 0.001
Distance to hospital in km	2.60	1.26	2.74	1.26	1.6	0.11

a Comparison used Yates' corrected chi-squared test for marital status, and Fisher's exact test for dichotomous variables.

b Poor education is defined by not achieving any ‘O’ levels, GCSEs or any higher education.

c Missing HADS data for 3 participants who had an emergency admission and 34 who did not.

d Maximum severity of all information provided. No information on severity in notes for 45 participants who had an emergency admission and 220 who did not.

e Comparison used t-test.

**Table 2:** Results of multiple logistic regression analyses with dependant variable, an emergency admission in the prospective year (data obtained from GP notes)

Possible risk factor	Model 1^a^			Model 2^a^		
Odds ratio	95% CI	Sig	Odds ratio	95% CI	Sig
No partner	1.55	1.05 to 2.28	0.027	1.49	1.04 to 2.15	0.032
IHD	1.66	1.10 to 2.52	0.016	1.60	1.04 to 2.46	0.033
Number of threatening experiences	1.14	1.03 to 1.26	0.011	1.16	1.04 to 1.29	0.008
Had an emergency admission in the previous year	–	–	–	3.41	1.98 to 5.86	< 0.001
HADS depression score of 8 or more	1.72	1.08 to 2.73	0.023	1.58	1.04 to 2.40	0.031

Age, sex, poor education, not working due to ill health, asthma, diabetes, COPD, cancer, stomach problems, high blood pressure, arthritis, distance to nearest hospital and maximum severity were included in the analysis, but were not significant, and not shown in the table.

Both analyses are adjusted for non-availability of data on emergency admissions in the prospective year using relevant sampling weights.

a Model 1 does not include emergency admissions in the previous year as an independent variable, whereas model 2 does.

**Table 3:** Odds ratios for emergency admissions in the prospective year for participants with HADS depression in 5 quintile groups

HADS depression score at baseline	Unadjusted			Adjusted^a^		
OR	95% CI	Sig	OR	95% CI	Sig
0–1	Reference group			Reference group		
2–4	1.36	0.78 to 2.36	0.28	0.99	0.52 to 1.85	0.96
5–7	2.43	1.44 to 4.12	0.001	1.73	0.94 to 3.18	0.078
8–10	2.25	1.31 to 3.87	0.003	1.67	0.87 to 3.21	0.12
11 or more	3.06	1.82 to 5.13	< 0.001	2.42	1.12 to 5.23	0.025

a Adjusted for age, sex, lack of partner, poor education, not working due to ill health, QOF diagnoses of diabetes, CHD, asthma and/or COPD, patient stated diagnoses of cancer, stomach problems, high blood pressure and/or arthritis, threatening experiences, distance from patient's home to the nearest hospital, and maximum severity of QOF illness and also adjusted for non-availability of emergency admission data using relevant sampling weights.

## Footnotes

1 The disparity between the number of patients consented and notes being reviewed is largely accounted for by the death of one of the GPs working in a single-handed practice and we were unable to retrieve the notes as patients had been registered in a number of practices in the city.
